# Fabrication of SiC Sealing Cavity Structure for All-SiC Piezoresistive Pressure Sensor Applications

**DOI:** 10.3390/ma14010128

**Published:** 2020-12-30

**Authors:** Lihuan Zhao, Haiping Shang, Dahai Wang, Yang Liu, Baohua Tian, Weibing Wang

**Affiliations:** 1 Institute of Microelectronics of the Chinese Academy of Science, Beijing 100029, China; zhaolihuan@ime.ac.cn (L.Z.); wangdahai@ime.ac.cn (D.W.); liuyang2@ime.ac.cn (Y.L.); tianbaohua@ime.ac.cn (B.T.); wangweibing@ime.ac.cn (W.W.); 2 Kunshan Branch, Institute of Microelectronics of Chinese Academy of Sciences, Suzhou 215347, China

**Keywords:** all-SiC, piezoresistive pressure sensor, room temperature bonding, bonding interface

## Abstract

High hardness and corrosion resistance of SiC (silicon carbide) bulk materials have always been a difficult problem in the processing of an all-SiC piezoresistive pressure sensor. In this work, we demonstrated a SiC sealing cavity structure utilizing SiC shallow plasma-etched process (≤20 μm) and SiC–SiC room temperature bonding technology. The SiC bonding interface was closely connected, and its average tensile strength could reach 6.71 MPa. In addition, through a rapid thermal annealing (RTA) experiment of 1 min and 10 mins in N_2_ atmosphere of 1000 °C, it was found that Si, C and O elements at the bonding interface were diffused, while the width of the intermediate interface layer was narrowed, and the tensile strength could remain stable. This SiC sealing cavity structure has important application value in the realization of an all-SiC piezoresistive pressure sensor.

## 1. Introduction

Nowadays, high temperature pressure sensors have attracted extensive attention and research [1,2,3], and are widely used in many industrial and engineering systems, such as turbine engines, oil well exploration, automotive fields [4,5] and measurement of pressure parameters in high temperature reaction vessels. Si (silicon)-based pressure sensors have reached the level of mature production and application; however, Si has limitations at high temperatures [6,7]. Subsequently, silicon on insulator (SOI) substrate significantly improves temperature resistance of the sensor [8,9]. However, SOI is still a Si substrate, and it is difficult to break through the bottleneck of its temperature resistance, which makes people focus on the realization and application of new materials and new technologies. In recent years, SiC has gradually come into people’s field of vision due to its excellent physical and chemical properties as well as high temperature resistance [10,11]. SiC is a very inert material because of its high bonding energy of Si–C bond, which makes it difficult to be oxidized, and it can withstand chemical corrosion and strong radiation damage in the external environment, and these features make SiC capable of excellent application potential in the environment of high temperature and strong corrosion, hence, all-SiC pressure sensors have been demonstrated to work at temperatures higher than 600 °C [12,13,14]. However, due to its high hardness, that is, three times that of Si material, and stable chemical properties, it is extremely difficult to process microelectronics and MEMS (Micro-Electro-Mechanical System) fabrication technology. For example, the preparation of the sealing cavity structure of the pressure sensor has always been a technological challenge for the realization of an all-SiC piezoresistive pressure sensor, as stated earlier, due to the high hardness and stable chemical properties of SiC. Whether by wet etching or dry etching, it is difficult to accomplish the deep etching of SiC (~300 μm) process, because deep etching will severely increase processing time (several hours), or is even impractical to conduct for hundreds of micrometers etching depth, also can cause scratches and poor uniformity of thin film thickness [12,15]. In the wet etching process, there is electrochemical corrosion [16], which is difficult to control with the etching rate and film flatness, and it is easy to cause lateral etching and shape distortion. In this regard, in recent years, many processing methods for SiC pressure sensor cavity structure have emerged, such as mechanical milling, laser scribing, ultrasonic drilling, etc. [17,18,19]. While these methods can improve the processing efficiency of the SiC cavity, it is difficult to ensure the flatness of the cavity surface and the uniformity of the diaphragm, which directly affect the working reliability of the pressure sensor and the overall performance of the device. 

**Figure 1:**
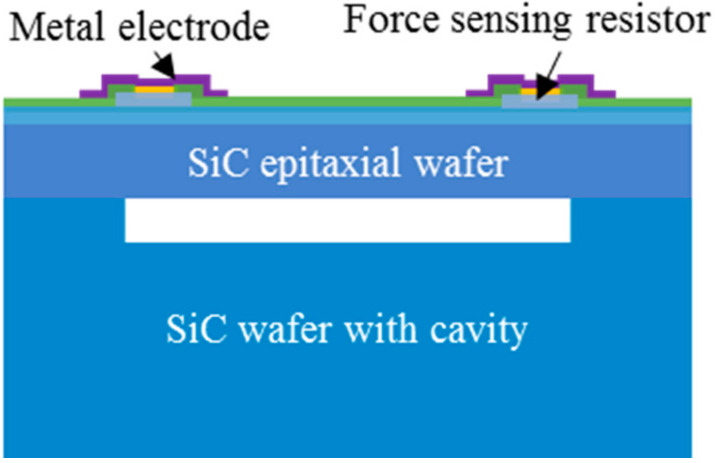
A simplified diagram of an all-SiC piezoresistive pressure sensor.

According to the above research status, we propose a processing method to achieve the sealing cavity structure for an all-SiC piezoresistive pressure sensor. The schematic diagram of the device is shown in Figure 1. In brief, a SiC diaphragm would be bonded onto a SiC wafer with shallow grooves using room temperature bonding technology (~23 °C), followed by force-sensing resistors and metal electrode fabrication. The force sensing resistors are located on the pressure diaphragm for sensing external test pressure and obtained by the plasma-etching process, and the electrode structure of the device is prepared through a metallization process. The structure is used for connecting the force sensing resistors with external test circuit. The SiC diaphragm is made by mechanical thinning and polishing the side of the nonepitaxial layer of the SiC epitaxial wafer. The SiC wafer with shallow grooves is obtained by inductively coupled plasma (ICP) shallow etching process (~20 μm). Notably, the shallow etching process of SiC can help avoid the time-consuming and laborious deep etching process and can obtain a cavity structure with a flat bottom surface. Moreover, the etch depth can be shallower, thus enabling the shallow grooves to function as overload protection during operation. The SiC thinning and polishing process is compatible with the existing semiconductor process, which is beneficial to improving wafer processing efficiency. In recent years, there has been much research and many breakthroughs on the SiC thinning and polishing process [20,21,22,23]. By optimizing the SiC thinning and polishing process, the whole wafer can achieve higher flatness and film thickness uniformity, so as to achieve the overall uniformity and consistency of hundreds of small diaphragms. For the method of SiC bonding, Yushin et al. have realized direct SiC bonding by loading pressure of 20 MPa in a vacuum environment of 800–1100 °C for 15 h [24] This method has the problem of high energy consumption. In addition, SiC bonding can use intermediate layers, such as Ni metal and glass, but the introduction of the medium layer can easily cause the mismatch of thermal expansion coefficients of different materials [25,26,27]. In our method, using a SiC–SiC room temperature bonding technology can help effectively avoid the material property mismatch problem and the energy consumption caused by a high temperature bonding process.

In previous research, we have successfully reduced the surface roughness of the outer cavity area, namely the bonding area, to less than 0.2 nm, by optimizing the process method of SiC cavity plasma-etching process [28]. The realization of this step optimization process has laid a foundation for the implementation of a SiC–SiC room temperature bonding process. In this work, we use the previously optimized etching process to prepare a cavity structure and utilize SiC–SiC room temperature bonding technology to bond the SiC cavity wafer with Si-face of an unprocessed SiC wafer instead of a SiC epitaxial wafer to form a sealing cavity structure. The experiment could serve as a demonstration of the proposal. By investigating a scanning acoustic image of the bonding interface and its morphology of the cross section, we determined that the two wafers were closely bonded. Furthermore, tensile strength of the bonding interface was tested, and the average bonding strength reached 6.71 MPa. It is well known that high temperature annealing is a necessary process for preparing device ohmic contact electrodes. The ohmic contact process of SiC usually goes through a high temperature environment of about 1000 °C for 1 min in N_2_ atmosphere. In order to further characterize the practicability and reliability of the sealing cavity, the bonded samples were subjected to a rapid thermal annealing (RTA) experiment at 1000 °C for 1 min in N_2_ atmosphere, and it was found that the intermediate transition layer was significantly narrowed. Furthermore, we extended the annealing time to 10 mins and found that the intermediate lattice became more ordered. It is essential that the bonding strength of the sample bonding interface was not affected by the two annealing conditions. The results proved the feasibility of the cavity application for the pressure sensor.

## 2. Experimental Procedures

**Figure 2:**
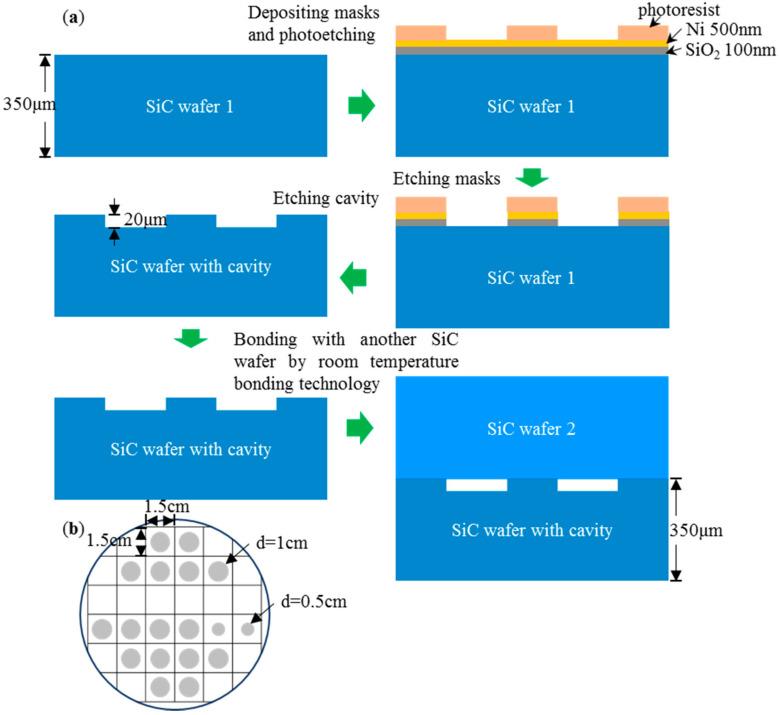
(**a**) SiC cavity fabrication procedure and modification process of bonding surfaces by Ar-FAB; (**b**) cavities distribution on the 4-in. bonding wafer.

Two pieces of 4-in. 4° off-axis 4H-SiC wafers with thickness of about 350 μm were used in this experiment, one as a substitute for a SiC epitaxial wafer, and one to attain SiC cavity structure through the ICP shallow etching process. The wafer surface roughness RMS (root mean square) is less than or equal to 0.2 nm by referring to the wafer parameter information. The cavity pattern is circular, and the diameter size was set as 1 cm and 0.5 cm. The patented wafer is shown in Figure 2b, no cavity structure was included. The cavity etching depth was about 20 μm. In our previous experiment, it was proved that when ICP etching uses conventional metal masks, after the mask is finally peeled off, there would be residual contamination in the area. However, this is very detrimental to the room temperature bonding process, since large roughness and particle containment could cause unbonded areas. So here, as shown in Figure 2a, we used SiO_2_ and Ni as the etching mask, according to our previous experiment method [28], which has optimized the roughness of the surface outside the cavity, that is, the bonding area, to less than 0.2 nm. First, a thickness of 100 nm SiO_2_ and 500 nm Ni were deposited on a SiC wafer. Second, the pattern of the cavity area was obtained by a photolithography process. After that, plasma-etching and wet etching were used for SiO_2_ and Ni layers, respectively, and then the SiC etching area was exposed. Subsequently, the SiC wafer was etched using SF_6_ and O_2_ gas by ICP-RIE (Reactive Ion Etching) etching process. Finally, SiO_2_ and Ni were corroded by the wet method. Detailed mask removal steps and cleaning methods can be seen in our previous study [28]. After the cavity wafer was obtained, an Ar-FAB beam was utilized in a supervacuum to remove contamination and oxide layer adhered on the two SiC surfaces. The wafers were then bonded to each other directly at room temperature under 20 MPa. Finally, the bonding wafer was cut into squares of 1.5 cm × 1.5 cm to facilitate the following tests, as shown in Figure 2b. Some of the small samples were annealed at 1000 °C in N_2_ atmosphere for 1 min and 10 mins, respectively.

In order to determine whether there are unbonded areas, the bonding interface was analyzed with scanning acoustic microscopy (SAM, PVA TePla, Wettenberg, Germany). Cross section interface morphology observation was characterized by scanning electron microscope (SEM, SU8200, Tokyo, Japan), transmission electron microscopy (TEM, FEI Talos, Hillsboro, OR, USA) and EDX line scanning analysis across the bonding interface before and after the annealing process. In addition, we tested the tensile strength of the bonded wafers by MFM1500 (TRY Precision, Shenzhen, China).

## 3. Results and Discussion

### 3.1. Characterization of Bonding Interface before Annealing

**Figure 3:**
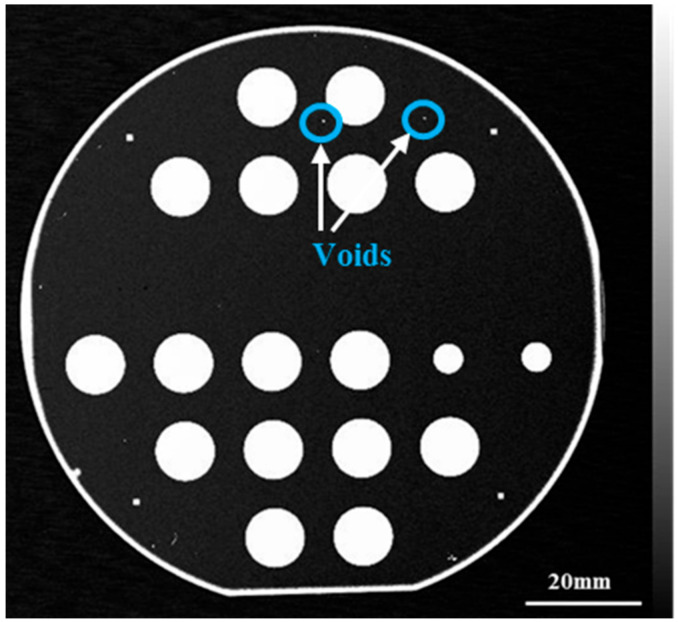
SAM image of the bonding wafer.

**Figure 4:**
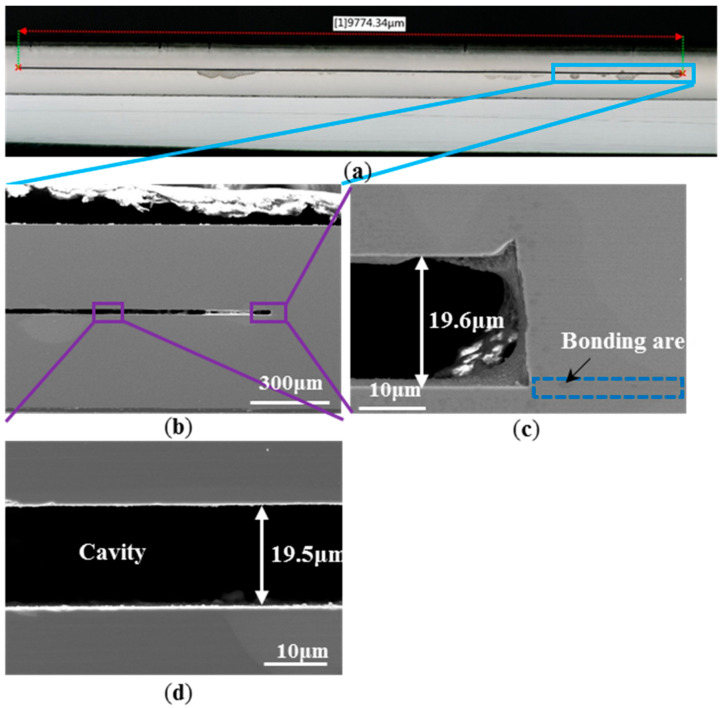
(**a**) Optical microscope image of the bonding interface cross section of the bonded sample with cavity diameter of 1 cm; (**b**) SEM image of the bonding interface section of the sample at low magnification; (**c**,**d**) are the local magnifications of the rectangular in (**a**).

Figure 3 describes the SAM image of the SiC bonded wafer, which shows that the majority of the wafer area was bonded very well except for sporadic little voids and the edge area. By analyzing the experimental process, the voids may arise from particle contaminations. Figure 4a is the optical microscope image of the bonding interface cross section of the bonded sample with a cavity diameter of 1 cm, and the measured diameter was 9774.34 μm. It is difficult to ensure that the sample is ground to the standard diameter, so there was some error from the standard diameter value of 1 cm. Figure 4b is the SEM image of the bonding interface section of the sample, adjacent images Figure 4c,d are the local magnification images. In Figure 4c, the blue dotted line box is the bonding interface area, where we cannot see obvious traces of the bonding interface. The measured cavity height was about 19.5 μm, which is consistent with the 20 μm depth of cavity etching in the ICP process. From Figure 4b, it can be seen that there was some contamination in the gap of the cavity, which was produced during the grinding and polishing of the sample cross section.

**Figure 5:**
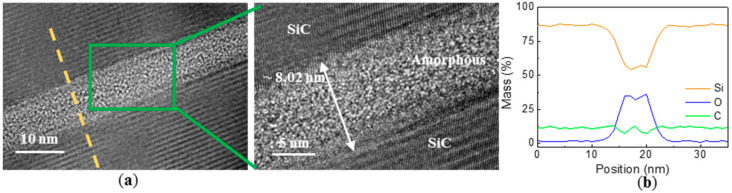
(**a**) Cross-sectional TEM micrographs of the bonding interface; (**b**) EDX line scanning analysis of the interface.

In order to further investigate the bonding properties of the bonding interfaces, a TEM cross-sectional image of the wafer bonding interface is illustrated in Figure 5a and its enlarged image, where no visible voids or cracks appear. At the interface, a bright seamless intermediate layer of about 8 nm should be amorphous because it has no lattice fringes and is distinct from adjacent crystalline phase. From data by previous studies, amorphous layer formation was caused by Ar-FAB bombardment [29,30]. Accordingly, EDX liner mapping of Si, C and O elements distribution of a small sample is shown in Figure 5b, the analysis location and range are roughly shown as the yellow dotted line in Figure 5a. It can be seen that there is a peak of O element at the bonding interface, because there was residual O element on the two wafer surfaces after the Ar plasma bombardment process. Additionally, Si and C spectra have remarkable troughs, the position of the troughs correspond to the position of the peak of element O, that is, the position of the intermediate layer of the bonding interface.

**Figure 6:**
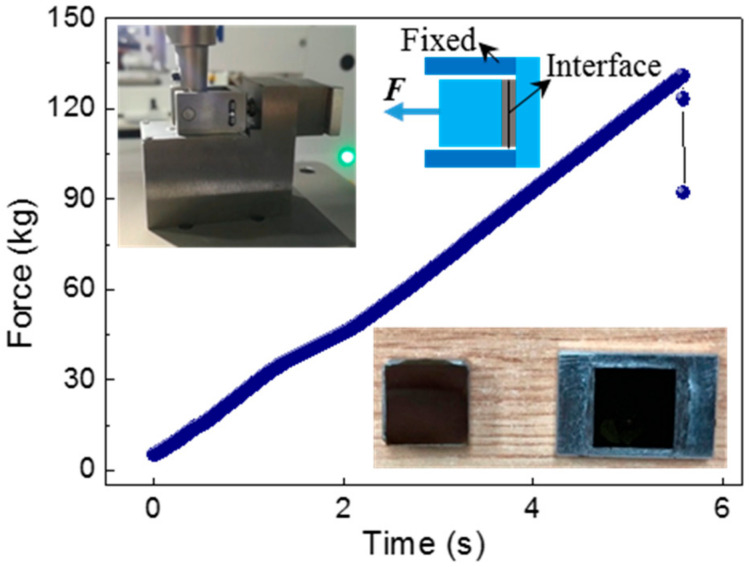
Tensile test curve of the bonding wafer. The upper left inserts show the physical and sectional view of the test fixture, and the lower right insert shows the wafer after separation.

To characterize the bonding strength of the bonding interface, a tensile test was performed. As shown in the illustrations in the upper left corner of Figure 6, the two illustrations are the physical image and section diagram of the fixture for the tensile test, respectively. Before the test, two metal blocks were glued to the two sides of the bonded sample, as shown in the illustration in the upper right corner of Figure 6. The metal block on the right was fixed by fixture, at which time a horizontal tensile force to the left was applied to the other metal block. As shown in the test curve in Figure 6, tension force increased with the increase of time, and the bonded interface separated at the moment of maximum tension, and the value was 130.838 kg. The tested sample was a chip with cavity diameter of 1 cm and its bonding area was 1.465 cm^2^. Tensile strength can be calculated by:

(1) $$\sigma_{tf}=\frac{F}{A}$$

where *F* is the maximum force that enables the bonding interface to cause separation, and *A* is total bonding area. Therefore, we could obtain a tensile strength of 8.75 MPa. The insert in the lower right corner of Figure 6 shows the appearance of the bonding interface after separation. We can see that the two surfaces after separation were relatively smooth, which means that the fracture surface of the sample basically occurred at the bonding interface or near it. In the same way, we tested eight samples including a large cavity, a small cavity and no cavity structure, and obtained an average value of 6.71 MPa. To our best knowledge, the minimum bonding strength recommended in MEMS device manufacturing is 4–5 MPa [31]. It is enough to show that the application of our experiment to the pressure sensor has very good feasibility.

### 3.2. Characterization of Bonding Interface after Annealing

**Figure 7:**
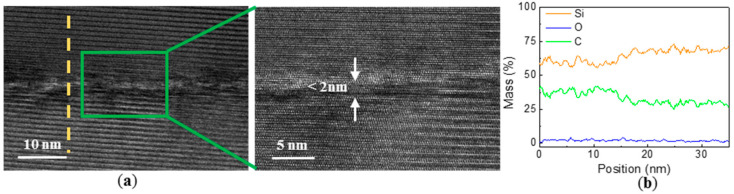
(**a**) Cross-sectional TEM micrographs of the bonding interface after annealing at 1000 °C for 1 min in N_2_ atmosphere; (**b**) EDX line scanning analysis of the interface.

Generally, ohmic contact structure should be prepared during the process of fabricating device electrodes. Conventional SiC ohmic contact preparation often needs to experience around 1000 °C high temperature environment for ~1 min. In order to prove the reliability of the cavity structure in the high temperature annealing process, two samples were placed in a rapid annealing furnace in N_2_ atmosphere of 1000 °C for 1 min. One sample was used to test the change of bonding interface properties, and the other sample was used to test tensile strength. Figure 7a shows the TEM image of the bonding interface after the sample underwent the RTA process. It can be clearly seen that the intermediate transition layer of the bonding interface became significantly narrower, almost less than 2 nm. In addition, tensile strength of another sample was measured at 9.33 MPa, indicating that the metallization annealing condition did not make bonding interface characteristics worse, instead, the width of the intermediate layer of the bonding interface was optimized. Figure 7b is the EDX analysis curve at the position marked by the yellow dotted line in Figure 7a. It can be seen that the fluctuation of Si, C and O elements at the middle interface layer disappeared, and O element diffused to the SiC side during the annealing process, C and Si element at the middle interface layer were also compensated. The reduction of intermediate thickness may be caused by densification of the intermediate layer and the outward diffusion of elements such as O.

**Figure 8:**
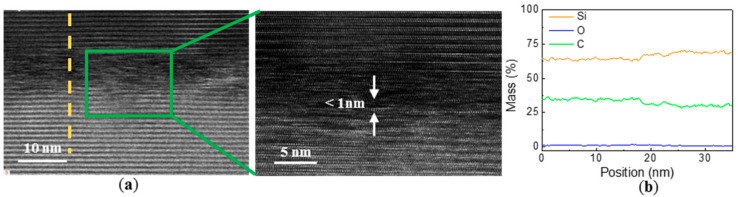
(**a**) Cross-sectional TEM micrographs of the bonding interface after annealing at 1000 °C for 10 min in N_2_ atmosphere; (**b**) EDX line scanning analysis of the interface.

Further, we increased the annealing time to 10 mins. In this experiment, TEM characterization and tensile strength testing were also performed for two samples. As shown in Figure 8a, the intermediate transition layer became narrower and fuzzier, with a width of almost less than 1 nm. Compared with the annealing experiment of 1 min, the proportion of O element seemed smaller, as shown in Figure 8b. This indicates that the diffusion of elements and densification degree of the intermediate layer become higher with the increase of annealing time. Via another sample, we obtained its tensile strength of 10.56 MPa. In brief, the diffusion of the element and the width of the interfacial layer were found by the RTA experiment. The sample can still maintain excellent bond strength after annealing.

## 4. Conclusions

Through SiC–SiC room temperature bonding technology combined with a SiC ICP shallow etching process, the preparation of a sealing cavity structure which can be applied to all-SiC piezoresistive pressure sensors was realized. SAM images demonstrate that the bonding surface interface produced only sporadic small voids. Moreover, the morphology and EDX line analysis of the bonding interface were characterized by TEM, which proves that the bonding interface was tightly bound. Average tensile strength of the bonding interface could reach 6.71 MPa. Through the experiment of RTA for 1 min and 10 mins in N_2_ atmosphere of 1000 °C, we found that Si, C and O elements diffused, and the width of the intermediate interface layer became narrower while maintaining excellent bonding strength, which indicates that the prepared sealing cavity structure had good stability in high temperature processing conditions, and the bonding interface could be optimized by increasing the high-temperature treatment time. As a result, the prepared SiC sealing cavity structure has great potential in the application of all-SiC pressure sensors.

## Data Availability

The data presented in this study are available on request from the corresponding author.
